# New insights of engineering plant exosome-like nanovesicles as a nanoplatform for therapeutics and drug delivery

**DOI:** 10.20517/evcna.2021.25

**Published:** 2022-06-29

**Authors:** Shafiu A. Umar Shinge, Yin Xiao, Jiang Xia, Yujie Liang, Li Duan

**Affiliations:** ^1^Department of Orthopedics, Shenzhen Key Laboratory of Tissue Engineering, Guangdong Provincial Research Center for Artificial Intelligence and Digital Orthopedic Technology, The First Affiliated Hospital of Shenzhen University, Shenzhen Second People’s Hospital, Shenzhen 518035, Guangdong, China.; ^2^Institute of Health and Biomedical Innovation, Faculty of Science and Engineering, Queensland University of Technology, Kelvin Grove Campus, Brisbane 4000, Australia.; ^3^Department of Chemistry, The Chinese University of Hong Kong, Shatin, China.; ^4^Department of Child and Adolescent Psychiatry, Shenzhen Kangning Hospital, Shenzhen Mental Health Center, Shenzhen 518020, Guangdong, China.

**Keywords:** Plant exosome-like nanovesicles (PELNVs), engineering exosomes, nanotheranostics

## Abstract

Plant exosome-like nanovesicles (PELNVs) are membrane-encapsulated nanostructures released from cells into their surroundings. PELNVs have an important role in intercellular and interspecies communication in all three domains of life. They act as protective compartments for the long-distance transit of signal molecules like proteins, nucleic acids, lipids, and other metabolites. A range of plants and vegetables can emit PELNVs. The importance of PELNVs in interspecies communication stems from their concentration in biomolecules (lipids, proteins, and miRNAs), lack of toxicity, ease of internalization by cells, and anti-inflammatory, immune-modulatory, and regenerative characteristics. PELNVs derived from numerous fruits and vegetables are biocompatible, biodegradable, and abundant in various plant species. Moreover, their convincing physicochemical characteristics underpin their modulative role in physiological and pathological processes, all of which have fueled speculation that these nanovesicles could be particularly adept at developing future-generation bio-therapeutic platforms. The goal of this review was not only to present an overview of the identified roles of PELNVs in physiology and pathology, but also to provide new insight toward their engineering for effective therapeutics and drug delivery nanoplatforms, a clue for future direction to the ongoing research gaps.

## INTRODUCTION

**Figure 1:**
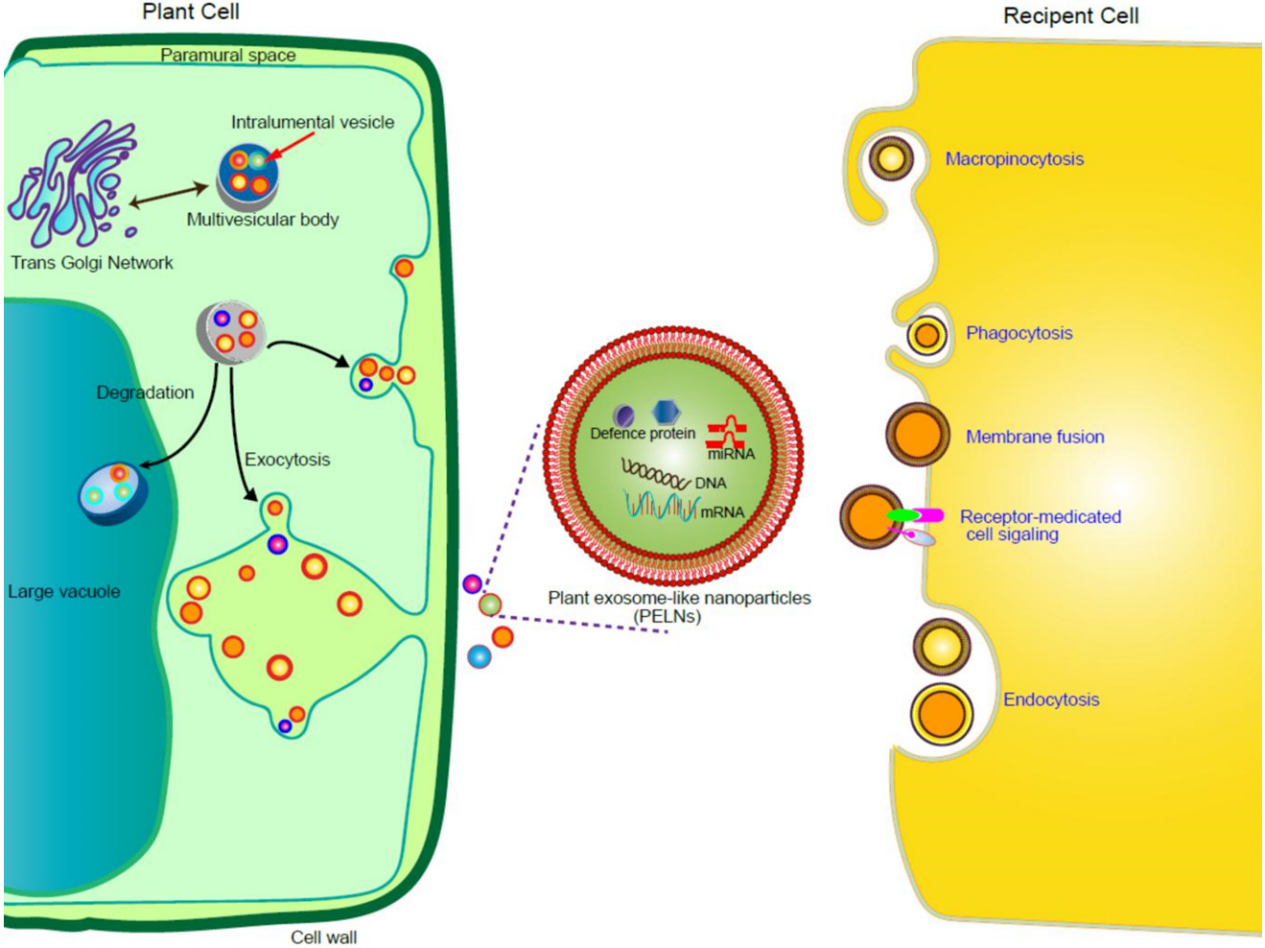
Schematic diagram of the biogenesis, release, structure, and uptake of PELNVs. PELNVs are formed by inward budding of the cell membrane and are produced by MVBs (also called late endosomes). The membrane of MVBs expands inwardly to fuse with the plasma membrane to release their intraluminal vesicles into the extracellular space (called exosomes) or fuse with lysosomes for degradation. In this process, proteins, nucleic acids (e.g., DNA, mRNA, miRNA), and lipid rafts are packed into PELNVs. A variety of mechanisms mediate the uptake of PELNVs, including the fusion of PELNVs with the cell membrane of recipient cells, resulting in the release of PELNVs cargo into the cytoplasm, uptaking by receptor-ligand interactions, endocytosis, and phagocytosis. PELNVs: Plant exosome-like nanovesicles.

Exosomes are extracellular vesicles with a 30-150 nm diameter secreted by eukaryotic cells. They are vesicles released from the cells after the fusion of intracellular multivesicular bodies (MVBs) [Figure 1]. After discovering exosomes in animal cells, more and more evidence shows that MVBs and plant exosome-like nanovesicles (PELNVs) appear in plants^[1,2]^. PELNVs have been isolated using different methods and show similar morphology and structure to animal exosomes. They have a phospholipid bilayer structure, contain proteins and microRNAs (miRNAs), and are saucer-shaped or cup-shaped can be observed with a transmission electron microscope (TEM). PELNVs are nanoscale particles released from various plants, including broccoli^[3]^, ginger^[4]^, grapefruits, and lemon^[5,6]^. They are crucial for normal cellular homeostasis, including control of the immune system, intercellular and interkingdom communications^[7]^, and involvement in physiological responses and pathological progression^[8]^. Moreover, their molecular constituents were correlated with numerous disorders and treatment responses, suggesting their potential for application as diagnostic instruments^[5]^. They are also employed in the development of tissue engineering and reconstructing, nucleic acids, and chemotherapeutical agent delivery, thus emerging as a novel form of nanomedicine.

Compared to synthetic nanocarriers, PELNVs are far better because they do not cause cell toxicity^[5]^. PELNVs also stimulate intestinal tissue renewal. Their exclusive lipid and miRNA content can modulate gut microbiota and play biological roles in fighting inflammatory disorders, including liver steatosis, colitis disease, and even cancers^[9]^. PELNVs can be loaded with therapeutic factors, including siRNAs, DNA expression vectors, proteins, and macromolecular therapeutics, and transferred to specific tissues in various disorders^[10]^. PELNVs can also be functionalized to deliver drugs into a target tissue conveniently. Despite all these promising features of PELNVs, the concept of molecular and cellular mechanisms accountable for their bio-functions is limited. However, the high pressure of interstitial fluid hinders homogeneous distributing and effective internalization of drugs in specific tissues such as a solid tumor. Functionalization of PELNVs nanocarriers could be an innovative approach for the delivery of drugs^[11,12]^.

Furthermore, poor loading and encapsulating efficacy and problems with exogenic hydrophilic macromolecules delivery and the possible delivery of undesirable cargo substances innately exist within PELNVs. This review discusses the recent development in PELNVs research and develops mechanistic insights for advancing their engineering for innovative therapeutic and drug delivery nanoplatforms. We propose that engineering the PELNVs, through an innovative approach for developing designer PELNVs with greatly enhanced and adjustable communication efficiency, is highly necessary to address these challenges. Thus, PELNVs can be offloaded, reengineered, functionalized, and reloaded with a cargo of choice or co-loaded with their contents without distorting their structural integrity to ensure higher loading and encapsulating efficacy cargo specificity and precision^[13]^.

### Engineering approaches for exosomes 

**Figure 2:**
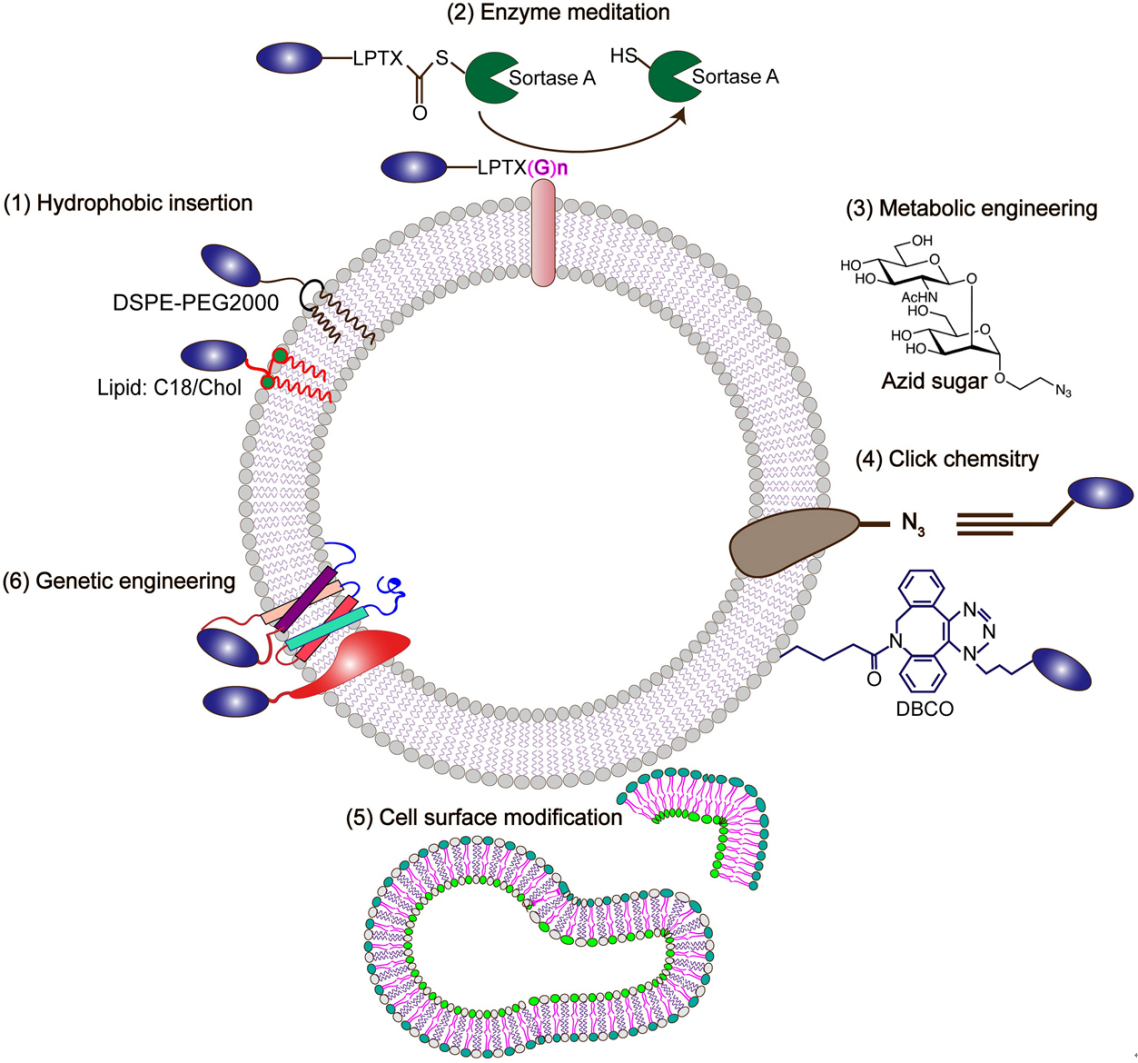
Reengineering PELNVs as nanoscale therapeutics. (1) Using lipophilic or amphiphilic molecules, these molecules can be directly inserted into the EV membrane through the hydrophobic interaction with the phospholipid bilayer. (2) Enzyme catalytic reaction, for example, sortase enzyme can react the sequence of LPETX with the N-terminal protein on exosomes. (3) Metabolic labeling, in which metabolite analogs are incorporated into cell biosynthesis, and functional groups (such as azide) can be introduced into EVs, thereby allowing subsequent bio-orthogonal reactions. (4) Chemical reactions can also be carried out directly on the vesicle membrane. For example, carbodiimide can modify natural amines to present azide groups for click chemistry reactions. (5) Exogenous substances can be introduced through liposomes or micelles fused with the exosomal membrane. (6) Genetic engineering can be used to fuse coding genes on exosomal membrane proteins.

Multiple disciplinary technologies have been developed for exosome engineering [Figure 2] and loading therapeutic cargos, including DNAs, RNAs, pharmacological agents, lipids peptides, proteins, and nanomaterials into exosomes^[14]^. Incubating, transfection, physical treatments like extrusion, electroporation, sonication, freeze-thawing, surfactant treatment, and dialysis, as well as in situ synthesis, are all employed^[10]^. Furthermore, molecular homing with substantial receptor affinities, acidic milieu, responsivity, and magnetic features has been assembled on exosomes by transfection or chemical modification, conferring the targeting capacity to exosomes.

### Indirect manipulation of exosomes

Plant-derived exosomes could be modified by genetic engineering of exosomes producing cells, including expressing fresh proteins on their membranes or increasing therapeutic proteins cargo load packing efficacy through selective peptides. In an animal model, a platelet-derived growth factor receptor transmembrane domain was fused to the GE11 peptide, which could selectively bound to EGFR on HEK293 cells. Subsequently, GE11 was found on HEK293 cell exosomes. These cells were then transfected with a synthetic let-7a miRNA. This let-7a containing exosomes could target tumor cells and efficiently distribute let-7a by interacting with EGFR and GE11 present on cancer cells. This administration method potentially affected treating EGFR-expressing breast cancer in a mouse model^[15]^.

### Direct manipulation of exosomes

Direct manipulation of exosomes is efficient for engineering exosome nanovesicles (ENVs) through direct modification^[16]^. A recent study proposed a way for directly conjugating biomolecules to the surface of ENVs using the click chemistry approach, which is a copper-catalyzed azide-alkyne cycloaddition. This work coupled alkyne groups to EVs via a copper-catalyzed azide-alkyne cycloaddition. This method did not affect EV size, adhesion, or internalization by recipient cells. Furthermore, this approach can successfully use bioconjugated micro-and macro-molecules onto the ENVs surfaces with various benefits, including excellent selectivity and compatibility to aqueous buffers. Another modified strategy for extending ENVs *in vivo* circulation period was also devised. This study transformed ENVs with EGFR conjugated to polyethylene glycol (PEG). A temperature-dependent transfer of nano-PEG-lipids to EV membranes was accomplished by mixing micelles with ENVs from neuro2A cells. The above modification method did not affect ENVs shape, size distribution, or protein content. Still, it extended EV circulation duration in an animal model, perhaps enhancing ENVs tissue-specific aggregation and cargo delivery efficacy^[17]^.

### Genetic engineering

Genetic engineering is a simple way of endowing exosomes with novel features. As in animal exosomes^[18,19]^, in plants, ligands or homing peptides fused with transmembrane proteins were found on the surface of exosomes. A recent study reported that the N-terminus of LAMP-2B is expressed on the surface of exosomes and may be attached with targeting sequences. Cell-specific binding peptides targeting specific organs or tissues can be screened and chosen by phage display and genetically changed at the N-terminus of LAMP-2B to achieve their targeting effects. Exosomes containing the designed peptide ligands are produced by cells that have been transfected with the plasmid. The TIWMPENPRPGTPCDIFTNSRGKRASNG peptide (TIWMPENPRPGTPCDIFTNSRGKRASNG) of the rabies virus glycoprotein (RVG) demonstrates preferential binding to acetylcholine receptors. Neuro-specific exosomes were produced to deliver pharmacological agents to the central nervous system (CNS)^[20]^. In the experimental mice, intravenous administered miRNA-124-loaded RVG exosomes entered ischemic cortical areas and induced neurogenesis. iRGD exosomes were also employed to precisely transport KRAS siRNA to v3-containing A549 tumors* in vivo*, resulting in specific KRAS gene knockdown and tumor growth reduction. In a recent study, we created chondrocyte affinity peptide (CAP, DWRVIIPPRPSA) modified exosomes that precisely transported miRNA-140 to chondrocytes in joints and slowed the progression of osteoarthritis^[21]^. Also, this genetic engineering technology was used to modify the exosomal membrane protein lamp2b to fuse with the mesenchymal stem cell affinity peptide E7, rending exosomes (E7-Exos) the ability to target mesenchymal stem cells. The modified E7-Exos can accurately deliver small molecular KGN into synovial fluid-derived MSCs (SF-MSCs), effectively promoting the differentiation of mesenchymal stem cells into chondrocytes, thus providing an advanced stem cell therapy for osteoarthritis^[22]^.

### Exosomal surface engineering

Exosomal surfaces could be efficiently manipulated, regardless of their origin. The most apparent purpose of surface engineering is to confer selective cell targeting. Genetic engineering and chemical managing are two manipulative technologies. Genetic engineering fuses gene sequencing to guided protein or polypeptide with a specified exosomal membrane protein^[23,24]^. This method is successful for peptide and protein surface display; however, it is restricted to targeting patterns that are genetically encoded. The chemical modification enables the presentation of a diverse spectrum of ligands, both natural and synthetic, through conjugation processes or lipid assembly. Conjugating reaction could covalently and stably transform exosomal surface protein. However, the complexity of the exosomal membrane might impair reaction efficacy and frequently limits the regulation of the response on a selective site^[25]^. Covalent manipulation might potentially compromise the vehicle's structure and functioning. Exosomes’ lipid bilayer can also be injected with lipids or amphipathic molecules, allowing their hydrophilic sections to be exhibited on the outside. This approach, driven by lipid self-assembly, might potentially increase exosomal toxicity.

### Exosome-like nanovesicles reengineering 

Preparing uniform-sized plant-derived exosomes is problematic since their size differs, ranging from 50 to 500 nm and even within species, posing a significant barrier to their application in the delivery of therapeutic drugs^[26]^. Furthermore, effective loading of drugs is a critical challenge that is difficult to achieve in pristine form in pure plant-derived exosomes^[27]^. As a result, it is vital to devise a method for consistently producing uniform-sized nanoparticles with adequate drug loading^[28]^. Researchers have successfully used the Bligh and Dyer technique, a well-known liquid-liquid extraction technique, to extract nano-lipids from plant-derived exosomes, which are then processed via a 200-nm liposome extruder reengineers the exosomes into equal-size nano-platform called plant derive exosomes nanocarriers^[29]^.

### Production of semi-synthetic exosomes by manipulation of natural exosomes through biotechnological engineering

Despite the biocompatibility and natural targeting capacity, plant-derived exosomes can be desirably manipulated based on targeted cells for efficient and specified therapeutic targets. This modification can be achieved through a process such as the integration of pharmaceuticals and other therapeutic agents and manipulating the surface charge for improved drug absorption. The most logical method of producing exosomes would be to harness the cellular machinery’s natural processes. The composition of exosomes at all levels is known to respond to a high level of regulation at particular cellular functions^[30]^. As a result, purposeful manipulation of the cellular environment might regulate the exosomal composition. When using this technology, exosomes might be created with a composition profile tailored to a given function. The selection of exosome-producing cells, including their *in vitro* harvesting settings, and the exosome isolation or enrichment processes, are critical components^[26]^. Plant-derived exosomes from several natural origins, including vegetable juice and fruits and secretions from mammals, have been manipulated earlier by different researchers to realize their potential for biomedical benefits in nanomedicine. Plant-derived exosomes could also be driven by internal modification. As discussed earlier, the exosome's cargos morphology is influenced by surface modification, whereby the exosomes' external surface morphology is manipulated to produce semi-synthetic plant-derived exosome nanovesicles^[31]^.

### Application of bio-engineered and simulated exosomes in nanomedicine

For larger therapeutic capacity, exosomes have been manipulated. This method sometimes includes introducing additional features for targeted purposes, *in vitro* or* in vivo* traceability, or administering the item^[30,32]^. In other circumstances, the goal of the manipulation was to improve colloidal stability or to adjust the surface charge to boost the rate of absorption^[4]^. These novel methodologies have given rise to new names such as bioengineered exosomes, artificial exosomes, exosome-mimetic nanovesicles, exosome-like nanovesicles, and exosome-based semi-synthetic vesicles^[33]^. These expressions have been used with many meanings in the literature, but there has yet to be a definite classification criterion. “Exosome-like vesicles” is used in certain research to describe artificial vesicles created from cells using various approaches to resemble exosomes. Other scientists, however, dubbed exosome-like nanovesicles with morphological and biochemical features comparable to exosomes. Other writers using non-animal research models adopted this name to describe vesicles with exosome-like size and flotation density values. For example, a prior study confirmed the presence of plant exosome-like vesicles in sunflower fluids, whereas another work detected exosome-like vesicles during pollen germination^[34]^. Several isolation procedures have been used to purify PELNVs for their application in nanomedicine [Table 1].

**Table 1:** Summary of the advantages and disadvantages of exosome isolation procedures

**Procedures **	**Purity**	**Principles**	**Sources**	**Advantages**	**Disadvantages**	**Reference**
Ultracentrifugation procedure	< 50%	Separations of constituents & solutes base on density and sizes	Ginger, grapefruit, carrot, grape, lemon, blueberry, shiitake, broccoli	Low cost & contamination risks, great yield & huge sample capacity	High cost, long run time, labor-intensive & low portability, potential damage by centrifugal speed	[35,36]
Size-based procedure	< 50%	Based on size among exosomes & constituents		Fast, direct RNA extraction, no special tool needed, good portability, high purity, maintains the integrity & biological activity, moderate sample capacity	Moderate purity, deterioration by shear stress, clogging potential and vesicle trapping, exosomes lost	[8]
Immunoaffinity capture-based procedure	99%	Fishing based on particular interaction among membrane receptors of exosomes & ligands		Best for isolation specificity, high purity, and subtyping potentials	High cost, low capacity, low yields, antigenic epitope might be blocked or masked	[27]

### Exosome cargo loading approaches

Several biomolecules are inherently enclosed in exosome-like nanovesicles. The content of exosomes and exosome-like particles may need to be redeployed to enhance their loading delivery and targeting efficiency^[37,38]^. Plant exosome-like particles contain higher biomolecules than animal exosome-like nanovesicles, although a redeployment approach could be introduced in cases where the bioactive component of interest is not present. Plant-derived exosome nanovesicles can be loaded with exogenous therapeutic molecules, including siRNAs, DNAs, proteins, and expression vectors, in addition to endogenous constituents, to ensure optimal therapeutic effects^[39]^. Various ways have been explored to load exosome-like nanovesicles with therapeutic compounds. These procedures begin with PELNVs from plants, which are then directly manipulated and loaded with pharmacological compounds. Active and passive cargo loading approaches are commonly utilized in the mechanical loading of exosome-like nanovesicles^[39]^. An incubation method is used in the passive drug loading procedure, in which plant exosome-derived nanovesicles are incubated with drug molecules at a specific temperature^[40]^. This drug encapsulation technique was driven by diffusion action and lipophilic contact between the drug molecule and the lipid bilayer of plant-derived exosomes. In addition to passive cargo loading, the sonication approach is applied, which disrupts the exosome-like nanovesicles membrane structure momentarily for successful cargo diffusion into exosome-like nanovesicles^[41]^. Following the cargo loading, the exosome-like nanovesicles' membrane morphology restores to its original state. This method was revealed to improve passive cargo loading by increasing cargo loading capability by up to 11 times. PELNVs retain a negatively charged surface in most situations, attracting oppositely charged chemical agents like doxorubicin and eventually facilitating drugs loading into their interior chamber using the sonication approach^[41]^. However, negatively charged molecules like FA and neutrally surfaced pharmacological agents like curcumin have been observed to get entangled in PELNVs regardless of modulating their biological activity. According to the findings, these molecules’ lipophilic features can lead to their encapsulation by overcoming the surface-related electrostatic force of nanovesicles. Because DNAs and siRNAs are very negatively-charged molecules, as shown by few reports, they may be loaded into PELNVs and perform as expected^[27,42]^. Nevertheless, compared to positively charged molecules, cargo loading efficiencies were deficient. The drug-loaded exosome-like nanovesicles are summarized in the table below [Table 2].

**Table 2:** Drug loaded PELNVs and their plant origin

**Pharmacological agents**	**Target cell/tissue**	**Source of nanovesicles**	**Reference**
miRNA, 6-shogaol & 6-gingerol	Intestinal epithelia	Ginger	[43]
	HT-29 cells & Colon-26 cells		
siRNA-CD98	Colon-26 cells & RAW 264.7 macrophages		[44-46]
Doxorubicin	Colon tumor cell line		[10]
Sulforaphane	Colon tissues	Broccoli	
Protein	Chronic myeloid leukemia	Citrus limon	[13]
miR-18a	Hepatic Kupffer cells	Grapefruit	
Luciferase gene siRNA, Paclitaxel, JSI-124 (anti-Stat3 inhibitor)	Cancer cells (GL26, 4T1, SW620, A549 & CT26)		
Curcumin & Doxorubicin	Colon tumors		[47]
Methotrexate	Intestinal macrophages		
miR17	Brain cancer cell line (GL26)		
miRNAs	Human epithelial colorectal adenocarcinoma cell (Caco-2)	Apple	[48]

### PELNVs in nanomedicine drug delivery system

**Figure 3:**
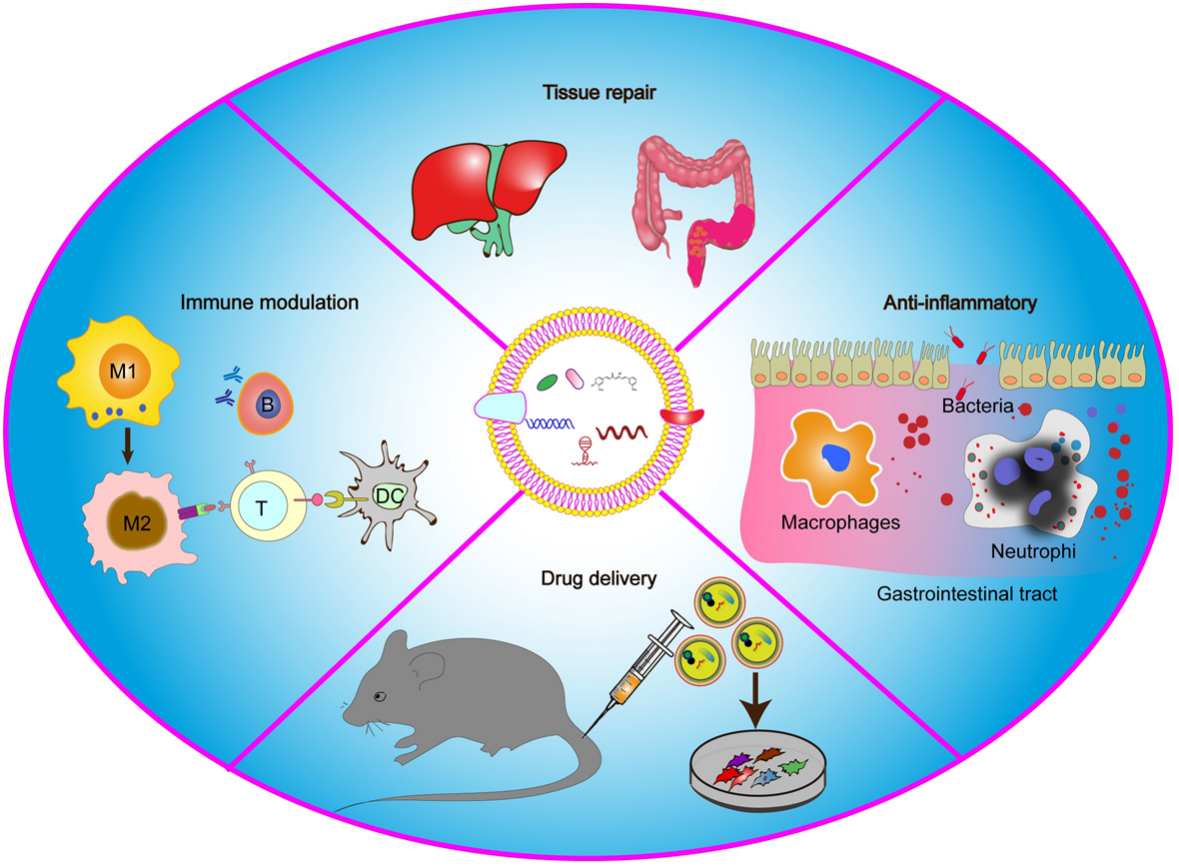
Therapeutic effects of PELNVs. PELNVs can regulate immunity and exert anti-inflammatory functions by inducing the functions of macrophages and dendritic cells *in vitro* and *in vivo*. PELNVs can deliver therapeutic reagents, siRNAs, and proteins to diverse cell types or *in vivo* animal models.

The realization of exosomes like nanovesicles as natural carriers of various biomolecules, including DNA, RNA lipids, peptides, and proteins, sparked apprehension that they could be employed to deliver exogenous molecules and therapeutic drug payloads. This strategy was proven by their ability to cross the blood-brain barrier (BBB)^[5,49]^. The brain was used as a targeted tissue because BBB is a significant barrier to the micro and macromolecular delivery of drugs to the CNS. Exosome-like nanovesicles are devoted to transporting payloads from nearby cells to distant locations and other species via kingdoms inter-communicating^[50]^. Their distinct shape and delivery capacity highlight their potential for application in the delivery of therapeutic drugs^[51]^. For the delivery of pharmacological agents, the benefits of plant-derived exosome nanovesicles-based nanoplatforms over synthetic nanoplatforms and mammalian-derived exosome-like nanovesicles have been established^[29]^. Exosome-like nanovesicles were initially used as a drug delivery nano-platform^[52,53]^. They require careful consideration since their inherent biological functions and origin play critical roles in their immunogenic acceptability. Exosome-like nanovesicles produced from mammalian malignant cells, for example, run the danger of spreading pro-malignant features to recipient cells [Figure 3]. Plant-derived exosome nanovesicles and artificially created nanoparticles such as liposomes and micelles share fundamental characteristics such as a similar lipid bilayers morphology and the capacity to carry both hydrophilic and hydrophobic payload.

Nevertheless, compared to chemically produced nanoparticles, plant exosome-like nanovesicles are biocompatible and have minimal immunogenic effects, more excellent cellular absorption, higher stability in the GI tract (GIT), and selective absorption targeting ability^[16]^. Furthermore, PELNVs-based nanoplatforms have a less complex creation procedure, whereas artificially generated nanoparticles such as liposomes necessitate complex manufacturing methods such as membrane extrusion, micro emulsification, etc. Furthermore, additional stringent modifications of synthetic nanoparticles for cargo loading and coating improvements, such as polyethylene glycol (PEG) coatings, are necessary to produce adequate immunological tolerance. However, PEG coating increases the circulation period and aids with immunological tolerance; nevertheless, these coatings may interfere with the interaction between the nanoparticle and targeted cells, reducing the biodistribution of the drug in the targeted tissue. Furthermore, repeated treatment with PEG-coated liposomes has been linked to the formation of anti-PEG antibodies, resulting in ineffective therapy^[54]^.

Furthermore, chemically synthesized nanoparticles only assist in the delivery of therapeutics but lack an inherent therapeutic advantage. Lastly, the lipid bilayer structure of PELNVs secures their cargo while avoiding enzymatic decomposition by proteinases and nucleases. These outstanding characteristics, combined with the discovery of their intrinsic therapeutic actions, make PELNVs an ideal candidate for entry into the field of drug delivery applications^[55-57]^ [Table 3].

**Table 3:** PELNVs for drug delivery nanoplatform

**Sources**	**Lipids**	**Targets**	**Modification**	**Loaded agents**	**Impacts**	**Reference**
Grapefruits	PC: 28.53% PE: 45.52%	4T1 breast tumor CT26colon tumor	Inflammatory receptors	Cur DOX	DSS-induced mouse colitis Cancer suppression	[6]
Ginger	MGDG: 18.9% PC: 6.5% DGDG: 27.4% PA: 41.9%	Female mice RAW264.7 Colon-26	Passed via 200nm polycarbonate membranes	siRNA CD98	Ulcerative colitis therapeutic benefits CD98 downregulation	[43,58]
Broccoli	High ratio of monoglycerides	C57BL/6 colitis mice	Unmodified	Sulforaphane	Colitis prevention in mice	[3,5]
Lemon	**-**	CT 26, SW620 colon tumor A549 tumor, ML LAMA84 CML xenograft mice SW480 Colorectal adenocarcinoma	Folic acid Unmodified	PTX Proteins	Tumor suppression Suppression of different cancer cell lines Bcl-xl, survivin, Bad, Bax, Tumor suppression suppression of angiogenesis, Bax, survivin, Bcl-xl	[59]
Grape	PE: 26.09% PA: 53.17% PC: 9.03%	Intestinal stem cells in Lgr5-EGFP -IRES-CreERT2 mice	Unmodified	No agent	Protect mice against DSS-evoked colitis Proliferation of Lgr5 intestinal stem cells	[60]

### PELNVs therapeutics application against diseases

The bioactivities of PELNVs have been demonstrated by earlier works, such as their regenerative function, lowering inflammation, promoting healing, reducing gingivitis, increasing the maturation of beneficial gut microbiota, and preventing cancer and infection^[8,44]^. Their biological processes in natural pristine morphological stability with intact bioactive payloads after simple separation from plants alleviate various pathologic problems in the kingdom of other species and provide different therapeutic options. Their plant origin and high safety profile of PELNVs and their various therapeutic potentials anchored in their active parent plants make them promising therapeutic candidates^[48,61]^.

### Concluding remarks 

Despite the infancy of PELNVs research, they have shown various advantages, including internalization, biocompatibility, cellular uptake, bioavailability, and targeting capability compared to their synthetic counterpart. Due to the excessive curiosity about PELNVs, there has been surplus interest in their potential application in disease treatment and therapeutic drug delivery settings^[62]^. Since they can mediate interkingdom communication, they prioritize nanomedicine for theranostics and cargo delivery applications. Various experiments have demonstrated exosomes to involve the exchange of substances between cells in physiology and pathology. Exploiting these exosome contents transfer mechanisms may prove crucial for advancing the engineering PELNVs with improved delivery and selective targeting. For example, the synthetic drugs and RNA delivery using polymeric nanomaterial, translocation to the nucleus is a significant issue because, while passaging RNAs could come across the lysosomal pathway, which induces the lysosomal substance degradation resulting in failure, and has a substantial effect on the delivery efficiency. However, engineered PELNVs can overcome this limitation. Thus, improving various aspects of PELNVs engineering through innovative nanotechnologies is crucial^[24]^. For such different innovative techniques required developing a novel strategy for exosome drug delivery, the significant challenges are the complexity of the extraction, the purifications, and storage. Further work is necessary to understand their biogenesis and the loading mechanisms for addressing these challenges. Developing a novel approach for long-term storage is also required for maintaining their integrity and bioactivities after purification.

## DECLARATIONS

### Copyright

© The Author(s) 2022.
